# The combined effects of tick defensin persulcatusin with conventional antibiotics and antimicrobial proteins/peptides against Staphylococcus aureus

**DOI:** 10.1099/mic.0.001589

**Published:** 2025-08-14

**Authors:** So Shimoda, Aoi Sugiyama, Megumi Onishi, Yusaku Tsugami, Yuya Nagasawa, Ryuta Tobe, Hiroshi Yoneyama

**Affiliations:** 1 National Institute of Animal Health, National Agriculture and Food Research Organization, 4 Hitsujigaoka, Toyohira, Sapporo, Hokkaido, 062-0045, Japan; 2 Laboratory of Animal Microbiology, Department of Animal Science, Graduate School of Agricultural Science, Tohoku University, 468-1, Aramaki Aza Aoba, Aoba-ku, Sendai, Miyagi, 980-8572, Japan

**Keywords:** mechanism of action, *Staphylococcus aureus*, synergistic effect, tick defensin

## Abstract

Persulcatusin (IP) is a tick defensin isolated from *Ixodes persulcatus* and is composed of 38 aa (molecular weight of 4,200). IP exhibits potent antimicrobial activity against *Staphylococcus aureus*, including drug-resistant strains, such as methicillin- and vancomycin-resistant *S. aureus*. Despite its potential use as an anti-*S. aureus* drug, its application remains underdeveloped because of several limitations, such as manufacturing costs and *in vivo* safety. Here, the combined effect of IP and other conventional antibiotics and antimicrobial proteins/peptides against *S. aureus* bacterial infections was investigated. Combinations of several antimicrobial compounds, including *β*-lactams, peptide antibiotics and lytic enzymes, showed a synergistic effect against *S. aureus* with a fractional inhibitory concentration index (FICI) of ≤0.75. In contrast, IP had an additive or irrelevant effect with a FICI of 1.0–2.0 when combined with several antibiotics such as chloramphenicol, gentamycin, kanamycin, erythromycin or vancomycin. Interestingly, *S. aureus* cells pretreated with IP for a short time demonstrated reduced susceptibility to daptomycin. Furthermore, it was determined that the mode of bactericidal activity of IP was substantially different in growth and non-growth states, suggesting that the mechanism of action of IP was associated with the inhibition of bacterial biosynthesis. These findings indicated that the combined effect of IP and conventional antibiotics has the potential to be used as an effective antimicrobial drug against *S. aureus*. Furthermore, it also suggested that an unknown mechanism of action of IP was associated with the inhibition of bacterial cell biosynthesis.

## Graphical Abstract

Tick defensin persulcatusin (IP) had a synergistic effect with conventional antibiotics and antimicrobial proteins/peptides. In contrast, IP reduced susceptibility to daptomycin, likely due to an overlapping target.

**Impact Statement**

Owing to the stagnation of antibiotic development and the emergence of drug-resistant bacteria, the production of novel antimicrobial agents is urgently required. Antimicrobial peptides (AMPs) are found in a diverse range of organisms and exhibit broad-spectrum activity against multiple pathogens, including drug-resistant bacteria. Arthropod AMPs, including tick defensins, have been isolated from various species worldwide. However, the application of these AMPs is difficult owing to their manufacturing costs and *in vivo* safety requirements. The combination of antimicrobial compounds is expected to reduce the amount used in clinical applications through synergistic effects, thereby contributing to the safe application of AMPs. This study demonstrated the synergistic effects of the tick defensin persulcatusin (IP) combined with conventional antibiotics as a model for the application of arthropod AMPs. Furthermore, the findings of this study indicate that IP reduced the susceptibility to daptomycin. Additionally, the bactericidal activity of IP against *Staphylococcus aureus* is related to the biosynthetic processes of the bacterial cell wall. These findings suggest that IP potentially interacts with an unidentified target molecule, such as precursor lipid II, which is involved in cell wall synthesis. This could potentially lead to the inhibition of cell wall biosynthesis in addition to its membrane-damage activity. This study contributes to the application of arthropod AMPs, which could lead to breakthroughs in the development of novel antimicrobial compounds.

## Data Availability

All relevant data are included in this article, and raw data not provided are available from the corresponding authors upon request. 

## Introduction

*Staphylococcus aureus* is a mammalian commensal bacterium and a pathogen. It typically colonizes skin and mucosal surfaces and causes a variety of pathogenic syndromes, such as abscess, bacteraemia, food poisoning and endocarditis [1,3]. Particularly, infections caused by multidrug-resistant *S. aureus*, such as methicillin-resistant *S. aureus* (MRSA), cause substantial morbidity and mortality in hospitals and community environments [4,5]. Currently, anti-MRSA drugs, such as vancomycin and daptomycin, are administered to treat MRSA infections [6]. However, there has been an emergence of *S. aureus* infections in hospitals that are resistant to both vancomycin and daptomycin [7,8]. Therefore, the development of novel antimicrobial drugs against *S. aureus* with a low probability of resistance emergence is paramount. Antimicrobial peptides (AMPs), isolated from various organisms, such as animals, plants and fungi, exhibit broad-spectrum activity against multiple pathogens, including bacteria, fungi and viruses [9,14]. AMPs are promising candidates for novel antimicrobial drugs due to their low probability of resistance evolution by rapid bactericidal activity [9,15, 16].

Persulcatusin (IP), a tick defensin isolated from the midgut of the hard tick (*Ixodes persulcatus*), is composed of 38 aa (molecular weight of 4,200). IP has potential as a novel antimicrobial agent with a potent and broad antimicrobial spectrum against Gram-positive bacteria such as *S. aureus*, *Bacillus subtilis* and *Corynebacterium* species, with antimicrobial activity equivalent to that of vancomycin and daptomycin [17,19]. Furthermore, IP also exhibited its bactericidal activity against drug-resistant *S. aureus*, such as MRSA and vancomycin-resistant *S. aureus* (VRSA) [17,18]. Previous studies have indicated that IP induces bacterial membrane disruption in *S. aureus* while demonstrating low haemolytic activity against mammalian cell lines, such as bovine colonic epithelial cells and bovine fibroblasts [18,20]. Furthermore, the emergence of mutants with tolerance to IP was comparable to that of vancomycin and less than that of nisin and daptomycin [19]. Despite the attractive characteristics of IP as a potential candidate for a novel antimicrobial drug, its clinical application has several limitations, such as *in vivo* safety (e.g. cytotoxicity and immunogenicity), unknown molecular mechanisms, limited understanding of structure-activity relationships and high manufacturing costs for peptide synthesis [16]. Therefore, the improvement of design and investigation strategies of IP application and other novel peptide derivatives with low toxicity has led to breakthroughs in their clinical application. However, information regarding safety, the mechanism of antimicrobial activity and *in vivo* resistant evolution of IP for clinical application is limited.

By combining antibiotics with other compounds, the antimicrobial spectrum can be broadened and dosage can be reduced [21], thereby potentially resolving the limitations associated with the clinical application of AMPs. Previous studies have reported synergistic activity of AMPs isolated from various organisms and conventional antibiotics against multiple pathogenic bacteria, including drug-resistant bacteria [22,24]. Furthermore, AMPs can also synergize with conventional antibiotics against multidrug-resistant bacteria, with the expectation of utilizing antibiotics that have been no longer in use due to concerns of the emergence of antimicrobial resistance [25]. Previously, arenicin-1 and HYL, antimicrobial peptides isolated from the marine polychaete lugworm *Arenicola marina* and solitary bee *Hylaues signatus*, respectively, exhibited synergistic activity with antibiotics against pathogenic bacteria such as *S. aureus* and *Pseudomonas aeruginosa*, thereby indicating their potential as antimicrobial agents [26,27]. Therefore, a combination of IP and conventional antibiotics is essential for the development of effective IP applications in clinical settings.

Therefore, this study aims to investigate the synergistic activity of IP against *S. aureus* in combination with conventional antibiotics, including *β*-lactams, peptide antibiotics (nisin and bacitracin) and anti-MRSA drugs (vancomycin and daptomycin). The findings will contribute to the development of IP as a novel antimicrobial agent against *S. aureus*.

## Methods

### Antimicrobial peptides and antibiotics

The antimicrobial agents used in this study are described in Table 1. Antimicrobial peptide IP (GFGCPFNQGACHRHCRSIGRRGGYCAGLFKQTCTCYSR) [17] and LL-37 (LLGDFFRKSKEKIGKEFKRIVQRIKDFLRNLVPRTES) [28] were chemically synthesized by Scrum, Inc. (Tokyo, Japan) and Genscript Japan (Tokyo, Japan), respectively. IP was dissolved in dimethyl sulfoxide at a concentration of 5 mg ml^−1^. The peptide solution was then diluted to a concentration of 160 µg ml^−1^ with sterile water and stirred aerobically to form disulphide bridges [29]. The products were analysed by ES-MS using a MicrOTOF-Q-II mass spectrometer (Bruker, Bremen, Germany).

**Table 1.** Antimicrobial agents used in this study

Compound	Class	Description	Company
Persulcatusin	AMPs	Tick defensin isolated from the hard tick	Scrum, Tokyo, Japan
LL-37	AMPs	Cathelicidin isolated from Human	Genscript Japan, Tokyo, Japan
Ampicillin	*β*-Lactam	Inhibitor of cell wall biosynthesis	FUJIFILM Wako Pure Chemical Corporation, Osaka, Japan
Cefazolin	*β*-Lactam	Inhibitor of cell wall biosynthesis	Tokyo Chemical Industry, Tokyo, Japan
Kanamycin	Aminoglycoside	Inhibitor of protein biosynthesis	Sigma-Aldrich, St. Louis, MO, USA
Gentamycin	Aminoglycoside	Inhibitor of protein biosynthesis	Tokyo Chemical Industry, Tokyo, Japan
Tetracycline	Tetracyclines	Inhibitor of protein biosynthesis	Sigma-Aldrich, St. Louis, MO, USA
Chloramphenicol	Phenicols	Inhibitor of protein biosynthesis	Sigma-Aldrich, St. Louis, MO, USA
Erythromycin	Macrolide	Inhibitor of protein biosynthesis	Sigma-Aldrich, St. Louis, MO, USA
Ciprofloxacin	Quinolones	Inhibitor of DNA biosynthesis	FUJIFILM Wako Pure Chemical Corporation, Osaka, Japan
Nisin	Lantibiotics	Membrane damage and inhibition of cell wall biosynthesis	MP Biomedicals, LLC, Irvine, CA, USA
Bacitracin	Polypeptide	Inhibition of cell wall biosynthesis	Sigma-Aldrich, St. Louis, MO, USA
Vancomycin	Glycopeptides	Inhibition of cell wall biosynthesis	FUJIFILM Wako Pure Chemical Corporation, Osaka, Japan
Daptomycin	Lipopeptides	Membrane damage and inhibition of cell wall biosynthesis	Tokyo Chemical Industry, Tokyo, Japan
Lysozyme	Lytic enzymes	Digestion of the cell wall	FUJIFILM Wako Pure Chemical Corporation, Osaka, Japan
Lysostaphin	Lytic enzymes	Digestion of the cell wall	FUJIFILM Wako Pure Chemical Corporation, Osaka, Japan

### Bacterial strains and media

*Staphylococcus aureus* subsp. *aureus* Rosenbach (ATCC 29213) (www.atcc.org) was obtained from the American Type Culture Collection (ATCC). Bacterial cells were cultured onto tryptic soy agar (TSA) (1.5 % tryptone, 0.5 % soy peptone, 0.5 % sodium chloride and 1.5 % agar) and Mueller–Hinton broth (MHB) (Becton Dickinson, MD, USA) at 30 °C. For the killing kinetics assay, bacterial cells were cultured in tryptic soy broth (TSB) (1.7% tryptone, 0.3% soy peptone, 0.5% sodium chloride, 0.25% glucose, 0.25% potassium di-phosphate and pH 7.4) or Luria-Bertani (LB) Lennox broth (1.0% tryptone, 0.5% yeast extract, 0.5% sodium chloride and pH 7.0) at 30 °C.

### Checkerboard assay

A checkerboard assay was performed to evaluate the combined effect of IP and conventional antibiotics using the broth microdilution method [30,31] with a final inoculum concentration of 5×10^5^ c.f.u. ml^−1^. Bacterial cells cultured in MHB at 30 °C were suspended in MHB or cation-adjusted MHB (caMHB) for daptomycin treatment. Bacterial suspensions were dispensed into 96-well round polypropylene plates (Watson Bio Lab, Tokyo, Japan) at an inoculum concentration of 5.0×10^5^ c.f.u. ml^−1^. The final concentration range of antimicrobial compounds was 0.25–16 µg ml^−1^ IP, 0.25–4 µg ml^−1^ ampicillin, 0.125–2 µg ml^−1^ cefazolin, 1–16 µg ml^−1^ kanamycin, 0.125–2 µg ml^−1^, gentamycin, 0.0625–1 µg ml^−1^ tetracycline, 1–16 µg ml^−1^ chloramphenicol, 0.0625–1 µg ml^−1^ erythromycin, 0.0625–1 µg ml^−1^ ciprofloxacin, 0.125–2 µg ml^−1^ vancomycin, 32–512 µg ml^−1^ nisin, 16–256 µg ml^−1^ bacitracin, 0.25–4 µg ml^−1^ daptomycin, 128–2048 µg ml^−1^ lysozyme, 0.25–4 µg ml^−1^ lysostaphin and 1–16 µg ml^−1^ LL-37. After incubation at 30 °C, the OD of each well was measured using a microplate reader (MTP-310; Corona Electric Co., Ltd., Ibaraki, Japan) at 660 nm (OD_660_). The MIC for each antimicrobial compound alone and in combination was determined based on the OD_660_ value, where growth was evaluated as negative when the OD_660_ was <0.05. The fractional inhibitory concentration (FIC) was calculated as the MIC of drug A in combination with drug B divided by drug A alone. The FIC index (FICI) was calculated as the sum of each FIC, and the lowest FICI for each combination was interpreted as follows: FICI of <0.5 was defined as synergy, FICI of ≥0.5 but <1 was defined as partial synergy, FICI of ≥1 but <4 was defined as an additive effect or irrelevant and FICI of ≥4 was defined as antagonistic [32].

### Killing kinetic assay

To examine the influence of *S. aureus* pretreated with IP on the efficacy of daptomycin against bacterial cells, a killing kinetic assay based on a modification of Grein’s method [33] was conducted. Bacterial cells grown in MHB to the exponential growth phase were incubated with 16 µg ml^−1^ IP (8×MIC) at 37 °C. Since IP interacts with the cell surface of *S. aureus*, causing depolarization within 15 min after incubation [19], the duration of the pretreatment with IP was set to 5 min. The cells were then centrifuged (5,000 ***g***, 5 min, 25 °C), and the supernatant was discarded to remove IP. Pretreated cells were resuspended in caMHB with or without 10 µg ml^−1^ daptomycin (10×MIC) and then incubated at 37 °C for 0, 15, 30, 60 and 120 min. The bacterial cells were then serially diluted with sterile PBS, and each dilution was plated on TSA plates. The colonies were left to incubate at 37 °C and were subsequently counted.

To study the antimicrobial effect of IP on *S. aureus* cells in both the growth and non-growth states, a killing kinetic assay of IP against *S. aureus* incubated in nutrient medium was performed as per the method described below or in the physiological buffer (PBS). Bacterial cells grown in TSB for ~24 h were adjusted to 1×10^8^ c.f.u. ml^−1^ in TSB, LB Lennox broth, MHB or PBS with 32 µg ml^−1^ IP or 16 µg ml^−1^ vancomycin, as control for antibiotic inhibited cell wall biosynthesis via interaction with lipid II, followed by incubation at 30 ℃ for a further 0.5, 1, 2, 4, 6 and 8 h. Bacterial cells were then serially diluted with sterile PBS, plated on TSA and incubated overnight at 37 °C. Colony counts were then determined, and c.f.u. ml^−1^ was calculated.

### Statistics

Statistical analyses were conducted using SPSS software (IBM SPSS Statistics version 25, Tokyo, Japan). Prior to statistical analysis, bacterial counts and survival rates followed a normal distribution as determined by the Shapiro–Wilk test. Statistical analyses were performed using one-way ANOVA, followed by Scheffé’s test to evaluate statistical differences. *P*<0.05 was considered statistically significant.

## Results

### IP exhibited a synergistic effect with conventional antibiotics

**Fig. 1.**
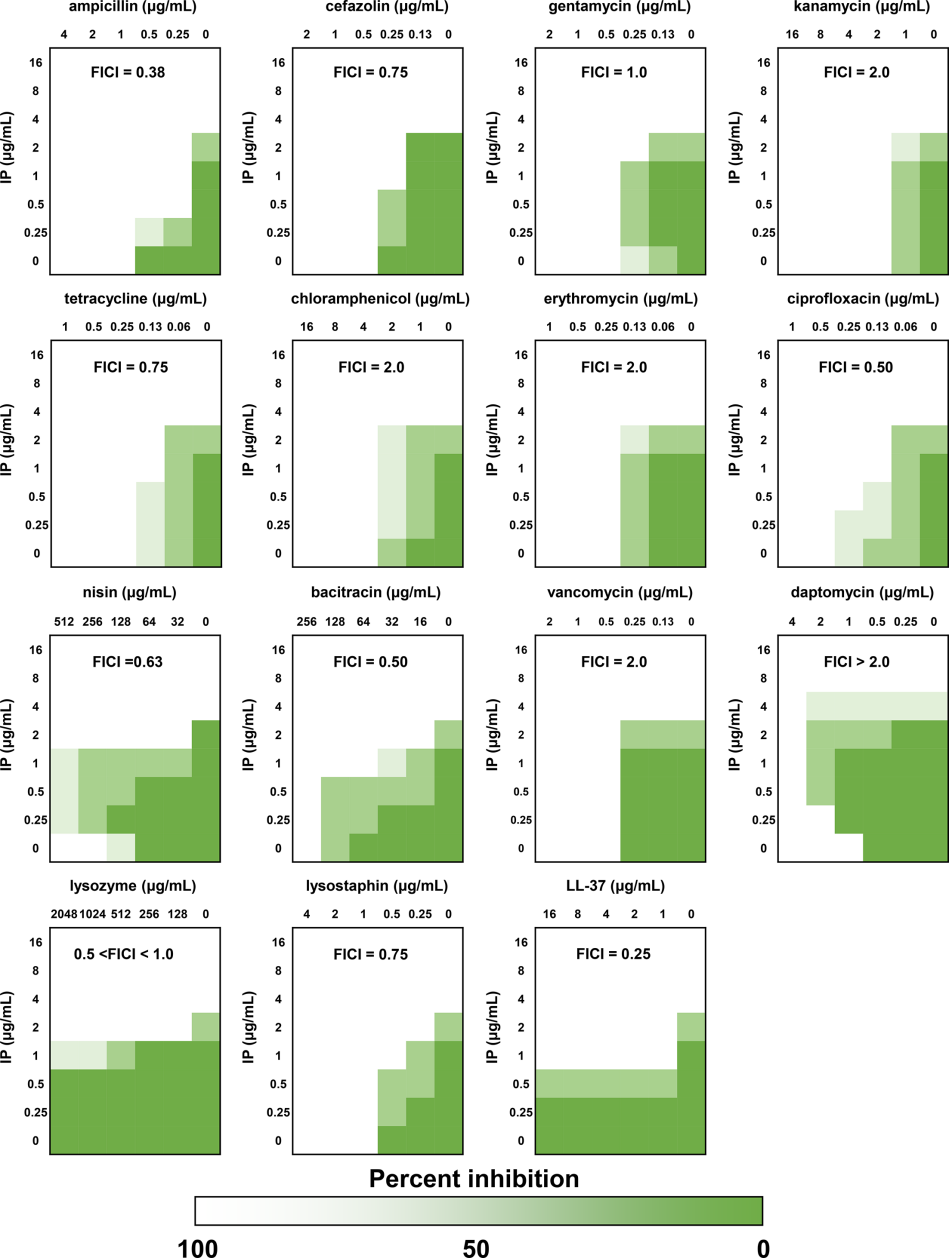
Heat plots of checkerboard assays of different conventional antibiotics and antimicrobial proteins/peptides combined with IP against *S. aureus* ATCC 29213. Green, growth; white, no growth. Synergy effect, FICI <0.5; partial synergy, FICI ≥0.5 but < 1; additive or irrelevant effect, FICI ≥1 but <4; antagonistic effect, FICI ≥4.

In the checkerboard assay, the combination of IP with ampicillin and LL-37 demonstrated synergistic effects with FICI results of 0.375 and 0.25–0.5, respectively, against *S. aureus* (Fig. 1). Partial synergistic effects were observed in combination with cefazoline, tetracycline, ciprofloxacin, nisin, bacitracin, lysozyme and lysostaphin with FICI of 0.5–1.0 (Fig. 1). Conversely, combination between IP and chloramphenicol, gentamycin, kanamycin, erythromycin or vancomycin showed an additive or irrelevant effect with FICI of 1.0–2.0 (Fig. 1). The FICI exceeded 2.0 only when combined with IP and daptomycin; the combination of IP with daptomycin was additive, irrelevant or antagonistic (Fig. 1).

### Pretreatment with IP reduced the susceptibility to daptomycin

**Fig. 2.**
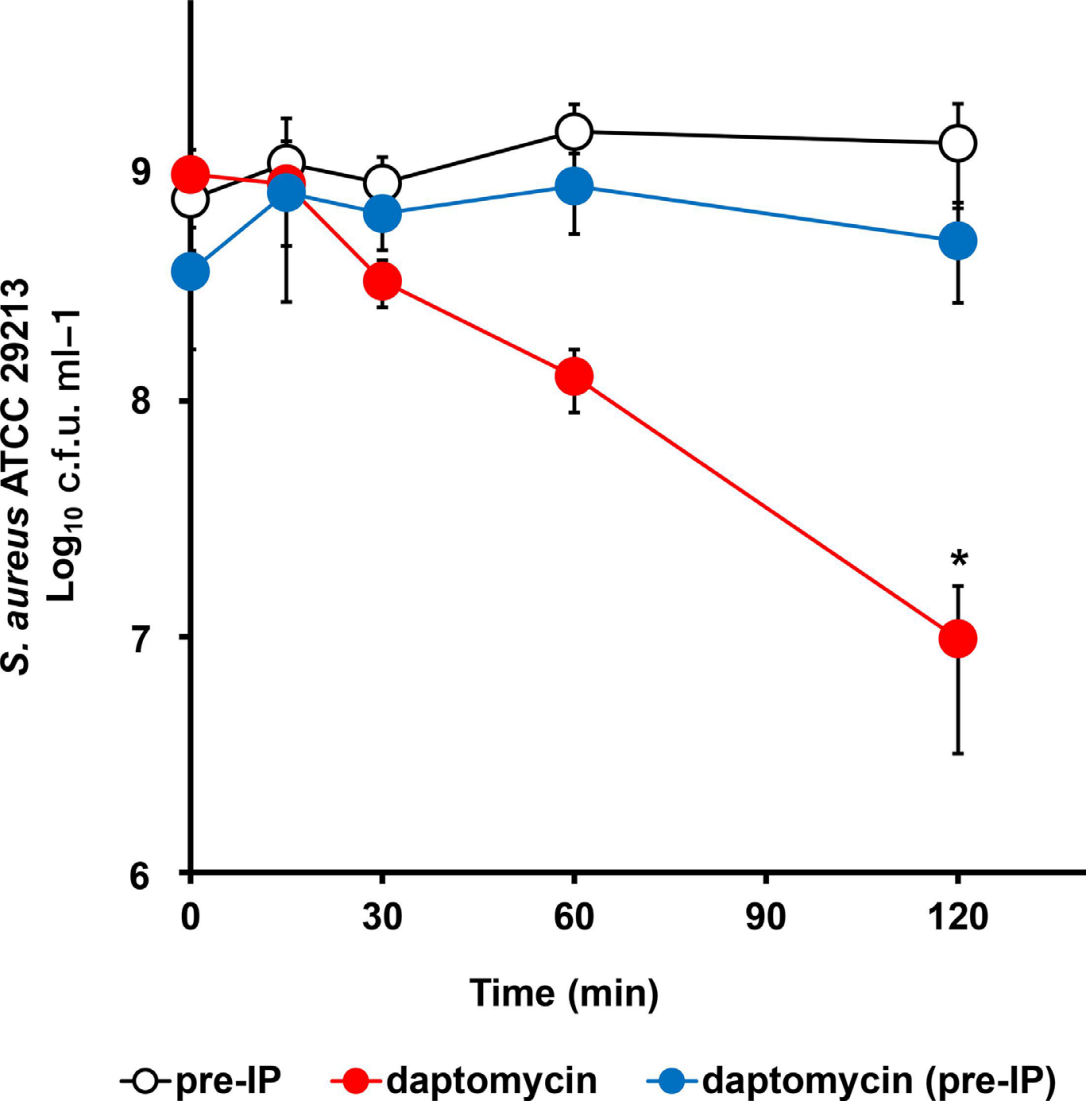
Killing kinetics assay. Daptomycin against *S. aureus* ATCC 29213 with or without pretreatment with IP (16 µg ml^−1^: 8×MIC). Red, daptomycin (10 µg ml^−1^: 10×MIC); blue, daptomycin with pretreatment of IP; white, pretreatment of IP. Each data point represents the mean value (± sd) of the corresponding triplicate. *P < 0.05 indicates statistically significant differences between groups with respect to the initiation time as determined by Scheffé’s test.

In the presence of sublethal concentrations of IP (0.25 to 1 µg ml^−1^ for nisin and 0.25 to 4 µg ml^−1^ for daptomycin), MICs of nisin and daptomycin were higher than those in the absence of IP (Fig. 1), implying that IP reduced susceptibility to nisin and daptomycin. These peptide antibiotics target cell surface components such as membrane phospholipids and cell wall precursor lipid II [33,36]; therefore, we hypothesized that the antimicrobial targets of IP overlapped with those of the antimicrobial agents or IP inhibited their antimicrobial activity. Thus, to reveal the overlapping of targets between IP and daptomycin, the effect of IP pretreatment on the bactericidal activity of daptomycin against *S. aureus* was further investigated using the methods of Grein *et al*. [33]. The bactericidal activity of daptomycin against *S. aureus* was potent and rapid. It resulted in a significant reduction in bacterial cells within 2 h from the initiation of the experiment (Fig. 2, *P*<0.05). Short-term pretreatment with IP (for 5 min) reduced the bactericidal activity of daptomycin; therefore, bacterial cells remained (Fig. 2).

### Bactericidal activity of IP

**Fig. 3.**
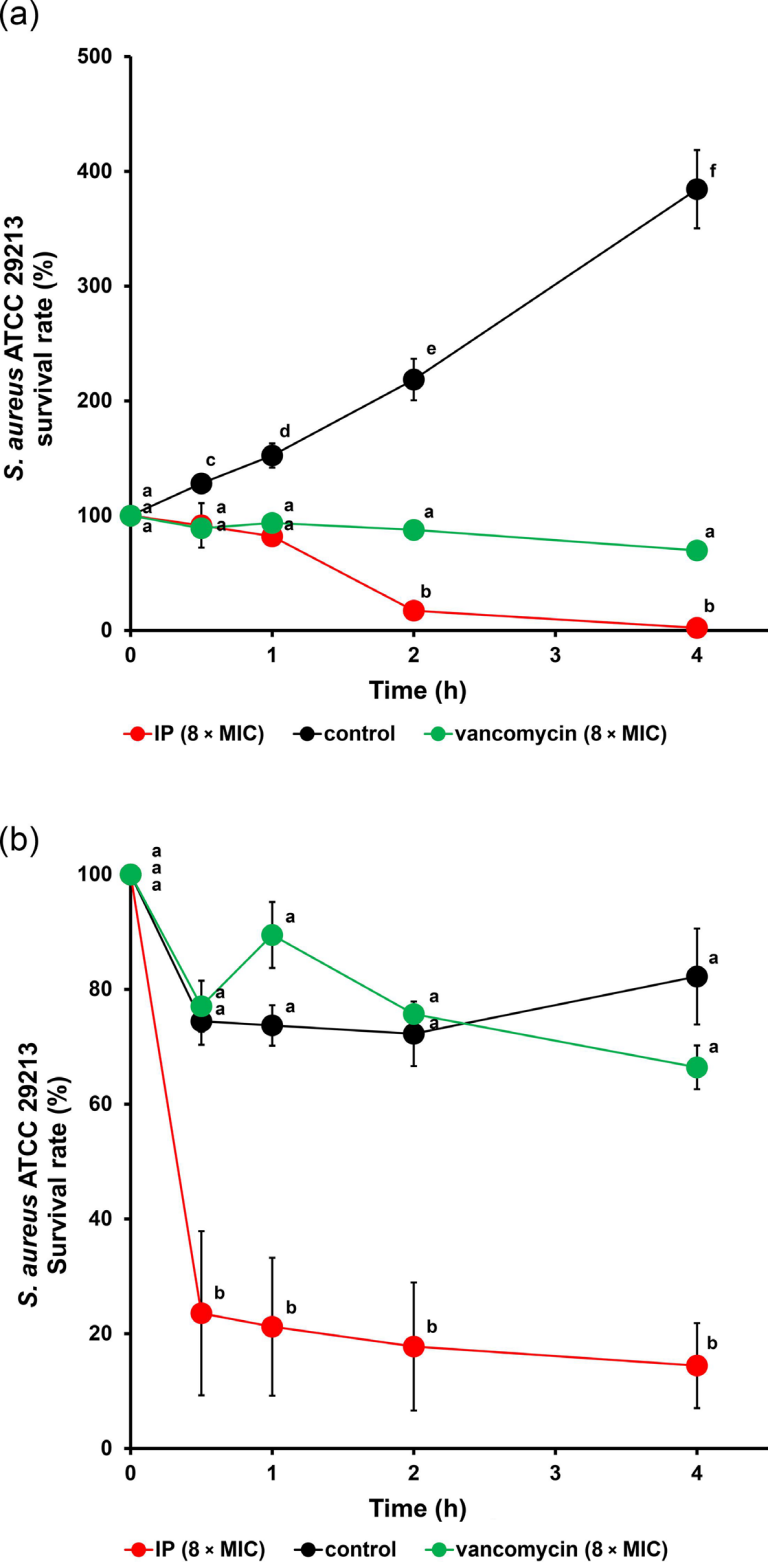
Killing kinetics assay. IP or vancomycin against *S. aureus* ATCC 29213 in the growth and non-growth states. Bacterial cells were incubated with IP (8×MIC, red), vancomycin (8×MIC, green) or without (control, black) in the growth state (TSB, a) or non-growth state (PBS, b). The survival rate was calculated as the percentage of the c.f.u. ml^−1^ at each time point divided by the c.f.u. ml^−1^ at the initiation time (0 h). Each data point represents the mean value (± sd) of the corresponding triplicate. Plots indicated with the same letters (a, b, c, d, e and f) were not significantly different at *P* < 0.05 as determined using Scheffé’s test.

It was hypothesized that IP has common target molecules, such as daptomycin, whose targets are bacterial cytoplasmic membranes and cell wall precursors [33,37, 38], implying that IP inhibits bacterial cell wall biosynthesis. Antimicrobial compounds that target cell biosynthesis require continuous cell wall biosynthesis to achieve bactericidal activity [39,41]. Therefore, the bactericidal activity of the IP against growth and non-growth cells was determined. IP exhibited bactericidal activity against growth cells (in TSB, LB Lennox or MHB) after 2 h in the early exponential growth phase (Fig. 3 and Fig. S1, available in the online Supplementary Material), in contrast, vancomycin induced a reduction of survival rate after 8 h in the late growth phase (Fig. S2). Notably, IP induced a significant reduction (*P*<0.05) in the survival rate (~70%) in the non-growth state within 0.5 h and then remained constant, though no reduction was observed in the treatments with vancomycin and only medium (Fig. 3b and Fig. S2).

## Discussion

AMPs have a broad antimicrobial spectrum and, therefore, have emerged as a promising class of bioactive compounds with the potential to be used as antimicrobial drugs against pathogenic bacteria. Currently, more than 4,000 AMPs have been isolated from animals, plants and fungi [9,10, 13, 14, 16, 25, 42]. However, there are limitations in the use of AMPs due to safety concerns or lack of tested efficacy [25]. Hence, AMPs are typically combined with other conventional antibiotics, and these combinations have demonstrated synergistic effects [22,23, 26, 27, 43, 44]. The synergistic effects of AMPs make it possible to reduce the concentration of antimicrobial agents used. IP, an AMP derived from *I. persulcatus*, has demonstrated potent antimicrobial activity against *S. aureus*. Furthermore, this AMP has a minimal influence on the development of resistant *S. aureus* [18,19]. Therefore, this study evaluated the combined synergistic effects of IP with other conventional antibiotics.

The findings of this study demonstrated that IP has synergistic or partial synergistic effects with conventional antibiotics such as *β*-lactams (ampicillin and cefazolin), ciprofloxacin and tetracycline (Fig. 1). *β*-Lactams, including penicillin, are important antibiotics that form the basis of medical practice and are currently among the most frequently used antibiotics [45]. Ciprofloxacin and tetracycline treat infections caused by Gram-negative and Gram-positive bacteria in hospitals [46,49]. Additionally, they are used in veterinary and agriculture, such as livestock production, to treat zoonotic diseases [49,50]. Hence, IP has the potential for combined applications with conventional antibiotics in various fields. Furthermore, IP combined with peptide antibiotics (nisin and bacitracin), lytic enzymes (lysozyme and lysostaphin) and human AMP (LL-37) showed synergistic effects against *S. aureus*. However, there is a lack of knowledge about the pharmaceutical characteristics and *in vivo* safety and the emergence of antimicrobial-resistant bacteria with the use of IP alone or in combination with antibiotics *in vivo*. It is essential to determine these basic data to effectively assess the clinical application of IP. The findings of this study demonstrated the potential of the combined application of arthropod AMP with conventional antibiotics, as well as peptide antibiotics and antimicrobial proteins/peptides, including lysozymes. Furthermore, this study is the first to report on the combined application of tick defensin and conventional antibiotics, with the expectation of the development of defensin family peptides isolated from various organisms.

The combination of IP with antibiotics that block bacterial protein synthesis, such as aminoglycosides (kanamycin and gentamycin), chloramphenicol and erythromycin, showed additive or irrelevant effects (Fig. 1). According to a previous study, we demonstrate that IP had antimicrobial activity against *S. aureus* cells, involving depolarization but not membrane permeability [19]. In contrast to this combined effect of IP, areucin-1 showed a synergistic effect with erythromycin and chloramphenicol against *S. aureus* (FICI of 0.375 and 0.5, respectively), which is consistent with the strong membrane permeability of areucin-1, resulting in enhanced uptake of these antibiotics [26]. No synergistic effect was observed between IP and antibiotics that function intracellularly, suggesting that IP did not enhance the uptake of these antibiotics. Furthermore, IP causes a strong reduction of membrane potential (depolarization) [35], which explains why IP does not show synergic effects with aminoglycosides since aminoglycosides are known to be internalized into bacterial cells by a self-promoted uptake mechanism using membrane potential [51]. Therefore, it is suggested that membrane depolarization by IP reduces the internalization of aminoglycosides, resulting in a non-synergistic effect between IP and aminoglycosides.

When IP was combined with nisin and daptomycin, the inhibitory effects of IP on the antimicrobial activity of nisin and daptomycin were observed (Fig. 1). The target of nisin and daptomycin is the bacterial cell wall precursor lipid II, which exhibits potent bactericidal activity via the inhibition of cell wall biosynthesis and membrane damage [33,36]. Furthermore, it was reported that some defensins, such as fungal defensin plectasin [52] and human *β*-defensin 3, inhibited cell wall biosynthesis via the interaction with lipid II [53,54]. The bactericidal activity of daptomycin against *S. aureus* pretreated with IP was significantly reduced compared to that against non-treated *S. aureus* (Fig. 2). Furthermore, short-term pretreatment with teixobactin, which targets lipid II, for 2 min reduced the efficacy of daptomycin against *S. aureus*, demonstrating potential overlap with daptomycin for its target molecule [33,38, 55]. Therefore, the mechanism of action of IP is speculated to be associated with that of daptomycin, i.e. the inhibition of bacterial cell wall biosynthesis via its interaction with lipid II. This finding is consistent with a previous study, where genes associated with IP resistance were also involved in nisin and daptomycin resistance [19,33, 35, 56].

For the development of derivatives and the effective application of antimicrobial compounds, it is essential to understand their precise mechanisms of action and target molecules. Accordingly, the bactericidal activity of IP in the growth and non-growth states, *S. aureus* cells was analysed to investigate whether the antibiotic activity of IP is associated with biosynthesis. Interestingly, IP showed a strikingly rapid bactericidal activity within 0.5 h in PBS; however, survival rates remained constant thereafter, though there was no bactericidal activity with vancomycin treatment, which inhibits cell biosynthesis (Fig. S2). The bactericidal activity of daptomycin against stationary *S. aureus* or a biofilm thereof was reduced more than in comparison to that in the exponential growth phase due to the accumulation of peptidoglycan and the change of membrane fluidity [57,58]. Thus, this rapid efficacy of IP may be represented by the state of the bacterial cell surface as it relates to the peptidoglycan and membrane order. Our previous reports showed that IP induced rapid membrane depolarization within 15 min against *S. aureus* cells [20]. This striking feature of IP could be the reason IP showed rapid bactericidal activity in the non-growth state via membrane damage, such as depolarization. In contrast, IP induced a significant reduction in the survival rate after incubation for 2 h (early exponential growth phase) in a nutrient medium, suggesting that the bactericidal activity of IP under these conditions could be caused by the inhibition of unknown biosynthetic pathway(s) (Fig. 3a). Daptomycin exhibits bactericidal activity against both growth and non-growth cells, damaging the bacterial membrane and interrupting bacterial cell wall biosynthesis via interactions with lipid II [33,39]. Moreover, a previous study showed that the mechanism of action of IP is partially similar to antimicrobial agents such as daptomycin, nisin and vancomycin, in terms of membrane damage and inhibition of cell wall biosynthesis [19]. Therefore, the mechanism of action of IP could include both membrane damage and inhibition of cell biosynthesis, which is similar to the mechanism of daptomycin and inhibition of cell biosynthesis, which is similar to that of vancomycin. However, the detailed mechanism of action of IP and the molecular biological evidence that it potentially has the same target as daptomycin, such as the membrane phospholipid and lipid II, remain unknown. In future research, it will be important to determine the molecular mechanism of action of IP and explore the antimicrobial activity of IP and its synergistic effect with conventional antibiotics against various *S. aureus* strains, including MRSA, to generalize the potential of IP as an anti-*S. aureus* drug.

In conclusion, this study demonstrated the combined effect of IP with conventional antibiotics and several antimicrobial proteins/peptides, suggesting the potential use of IP as an anti-*S. aureus* drug. Furthermore, it showed that IP reduced the susceptibility to daptomycin and exhibited bactericidal activity against *S. aureus’*s ongoing biosynthesis in a nutrient medium. These results suggest that IP has a mechanism of action associated with inhibition of cell biosynthesis, with the same target as daptomycin, such as lipid II. This study contributes to the novel application of AMPs isolated from arthropods, which are the most diverse species on Earth, and also reveals the complex mechanisms of action of AMPs.

## Supplementary material

10.1099/mic.0.001589 Uncited Supplementary Material 1.

## Abbreviations

AMPs: antimicrobial peptides
ATCC: American Type Culture Collection
caMHB: cation-adjusted MHB
FIC: fractional inhibitory concentration
FICI: fractional inhibitory concentration index
IP: persulcatusin
LB: Luria-Bertani
MHB: Mueller–Hinton broth
MRSA: methicillin-resistant *Staphylococcus aureus*
TSA: tryptic soy agar
TSB: tryptic soy broth
VRSA: vancomycin-resistant *Staphylococcus aureus*
