# Supplementary figures and images for: The combined effects of tick defensin persulcatusin with conventional antibiotics and antimicrobial proteins/peptides against Staphylococcus aureus
Source: Microbiology (Reading). 2025 Aug 14;171(8):001589. doi: 10.1099/mic.0.001589 (PMC12453121; doi:10.1099/mic.0.001589)

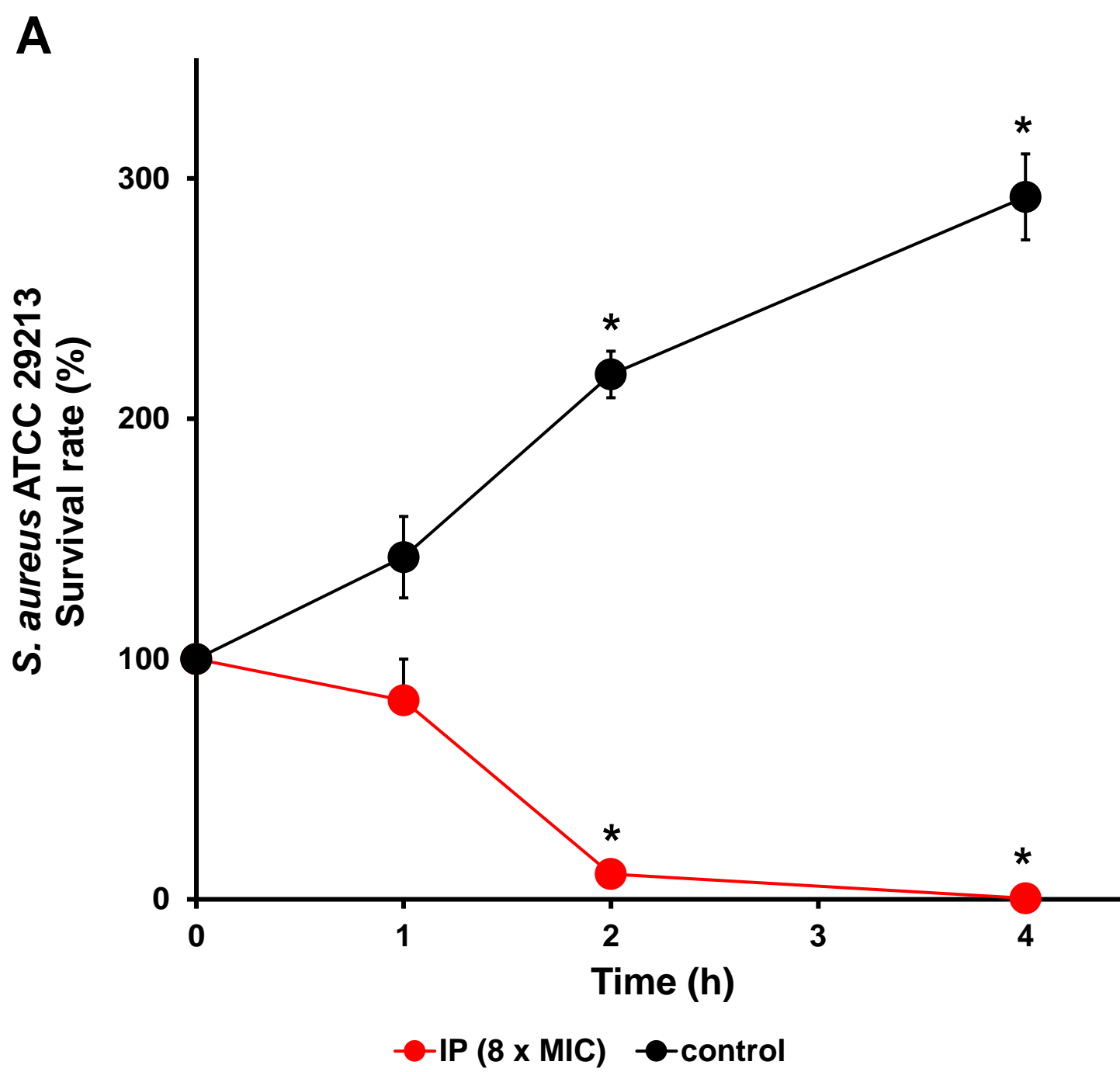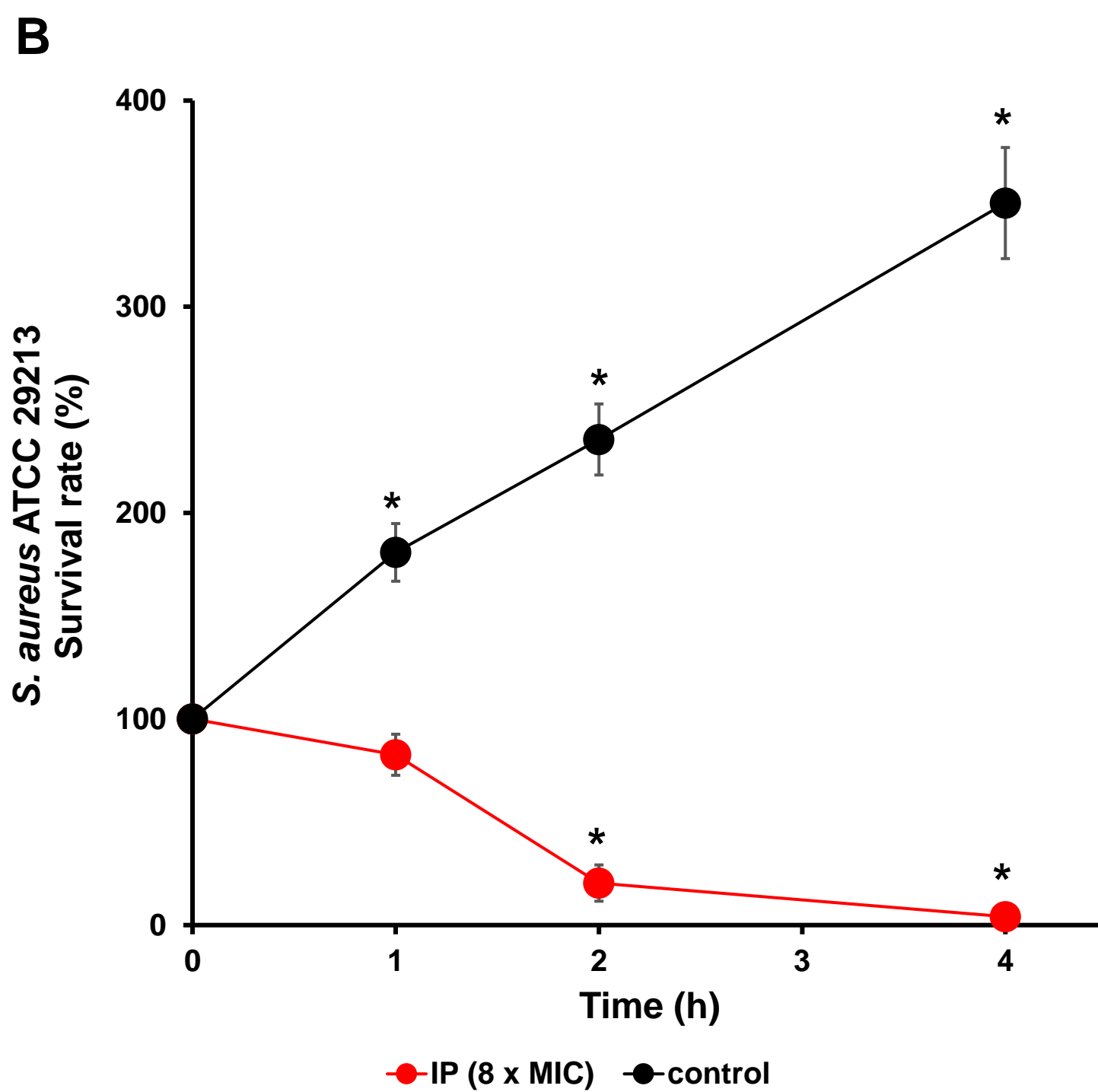

Supplemental Figure 1

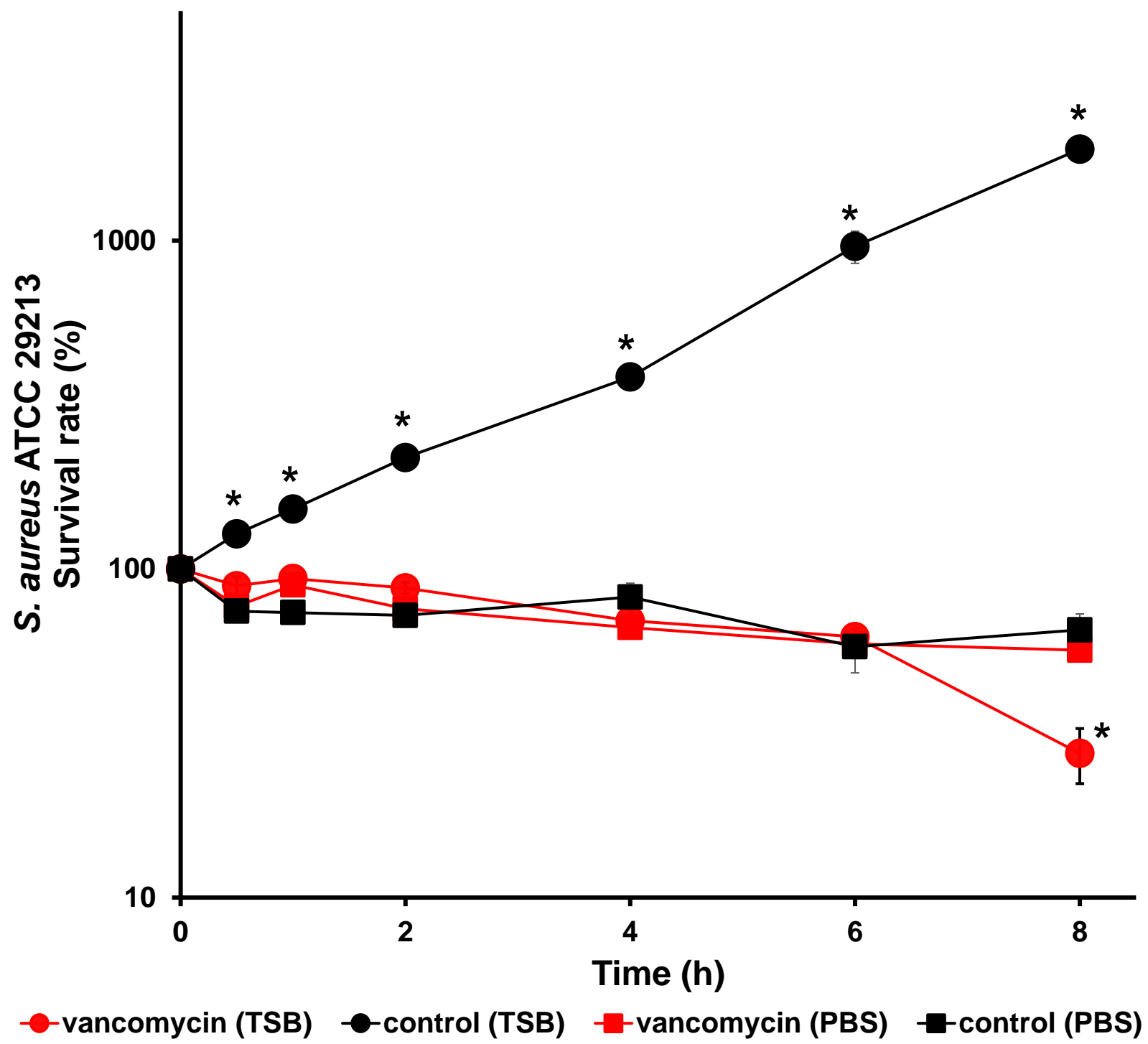

Supplemental Figure 2

Supplement: Uncited Supplementary Material 1. [file mic-171-01589-s001.pdf]
